# Translation from a Paper

**Published:** 1883-01

**Authors:** 


### Translation from a Paper.

Koch has already identified the capability of resistance of the bacteria against most of the ordinary disinfectants. Recently Frankland has studied the behavior of the bacteria to different gases and he found they were able to live under circumstances which would destroy higher organisms in the shortest time. He took some broth and put some seed of bacteria to it, closed them up in a glass over mercury and then introduced various gases. Oxygen, hydrogen, nitrogen, and carbonic acid made not the slightest impression, and even when after introducing sulphuric acid the health of the little organisms seemed very little affected, they were as lively as before. When cyanogen was introduced they appeared somewhat feeble, but in course of a week recovered completely. According to this investigation there seems to be very little prospect to destroy bacteria by means which the human body can bear.

